# Effective silicon production from SiCl_4_ source using hydrogen radicals generated and transported at atmospheric pressure

**DOI:** 10.1080/14686996.2020.1789438

**Published:** 2020-07-27

**Authors:** Yuji Okamoto, Masatomo Sumiya, Yuya Nakamura, Yoshikazu Suzuki

**Affiliations:** a Widegap Semiconductor Group, National Institute for Materials Science, Tsukuba, Japan; b Graduate School of Pure and Applied Sciences, University of Tsukuba, Ibaraki, Japan; c Faculty of Pure and Applied Sciences, University of Tsukuba, Ibaraki, Japan

**Keywords:** Hydrogen radical, Siemens method, W-filament, SiCl_4_, 301 Chemical syntheses / processing

## Abstract

In the Siemens method, high-purity Si is produced by reducing SiHCl_3_ source gas with H_2_ ambient under atmospheric pressure. Since the pyrolysis of SiHCl_3_, which produces SiCl_4_ as a byproduct, occurs dominantly in the practical Siemens process, the Si yield is low (~30%). In the present study, we generated hydrogen radicals (H-radicals) at pressures greater than 1 atm using tungsten filaments and transported the H-radicals into a reactor. On the basis of the absorbance at 600 nm of WO_3_-glass exposed to H-radicals in the reactor, we observed that H-radicals with a density of ~1.1 × 10^12^ cm^−3^ were transported approximately 30 cm under 1 atm. When SiCl_4_ was supplied as a source into the reactor containing H-radicals and allowed to react at 850°C or 900°C, Si was produced more efficiently than in reactions conducted under H_2_ ambient. Because the H-radicals can effectively reduce SiCl_4_, which is a byproduct in the Siemens method, their use is expected to increase the Si yield for this method.

## Graphical abstract

**Figure:**
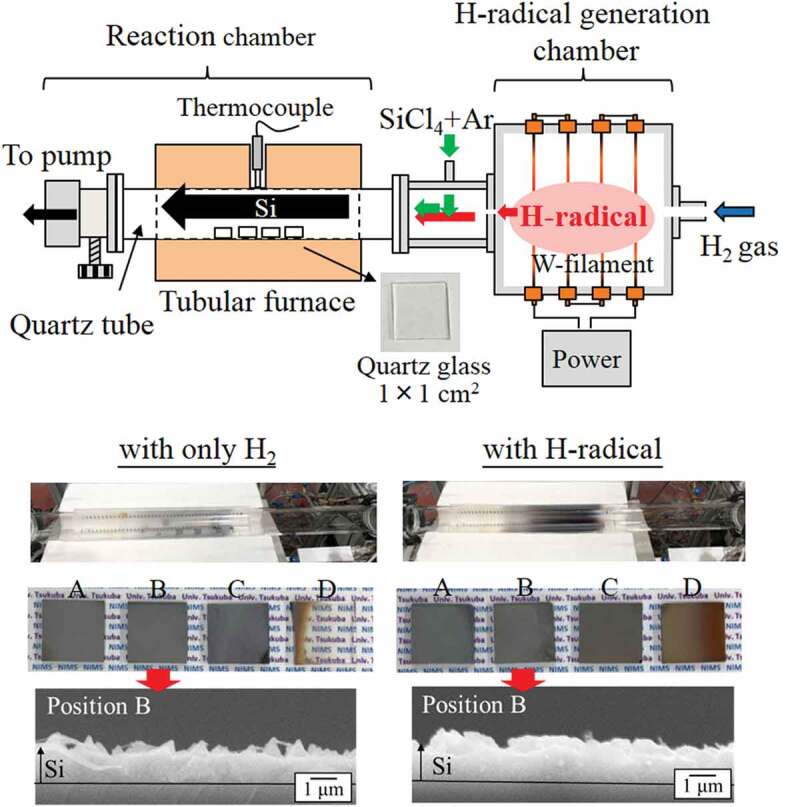

## 1. Introduction

Si is regarded as strategic and important material because it is a necessary component of computers, motor vehicles, and various household appliances. The realization of a sustainable society necessitates proper utilization and management of natural energy resources. Photovoltaics (PVs) is one of the most promising technologies for achieving this objective. Although various materials (e.g., chalcogenides, III–V compound semiconductors, hydrogenated amorphous Si, dye-sensitized anode materials, perovskite-structured compounds, and organic materials) have been used in PV devices, 90% of such devices are based on crystalline Si. For PVs to contribute substantially to worldwide energy production by 2050, 1 TW of PV energy production capacity must be installed per year [1]. From the viewpoint of material resources, 1 TW corresponds to ~1000 tons of crystalline Si. Huge amounts of high-purity Si are required to support daily life, which relies heavily on the use of electronic devices and the consumption of large amounts of energy.

High-purity Si has been used as the raw material for Si solar cells and semiconductors. High-purity Si can be categorized into semiconductor-grade Si (purity: ~11 N) and solar-grade Si (purity: ~6 N). Demand for high-purity Si has recently increased because of an increase in the amount being used in the production of PV solar cells. Among the methods used to produce high-purity Si, the Siemens method is the most popular and has been widely used [2,3]. In the Siemens method, high-purity trichlorosilane (SiHCl_3_) and H_2_ gas are introduced into a bell jar; the SiHCl_3_ is then reduced by H_2_ into Si on heated Si rods at approximately 1000–1200°C at 1 atm (eq. 1):

(1) $$SiHCl_{3}\left(g\right)+H_{2}\left(g\right)\to Si\left(S\right)+3HCl\left(g\right)$$

However, in the actual process, the pyrolysis of SiHCl_3_ dominantly proceeds instead of the reduction because the Gibbs free energy (Δ*G*) of the pyrolysis reaction is lower than that of the reduction reaction [4]. The SiHCl_3_ pyrolysis reaction produces 1 mol of Si and 3 mol of SiCl_4_ (byproduct) from 4 mol of SiHCl_3_ (eq. 2):

(2) $$4SiHCl_{3}\left(g\right)\;\to 3SiCl_{4}\left(g\right)+Si\left(s\right)+2H_{2}\left(g\right)$$

Hence, the Si yield of the SiHCl_3_ pyrolysis reaction is only ~25%. In addition, Si can’t be produced from hydrogen reduction of SiCl_4_ because it is more stable than SiHCl_3_ [4]. Consequently, the Si yield of the Siemens method is limited to ~30%.

To improve the Si yield of the Siemens method, we have focused on hydrogen radicals (H-radicals) as a reducing agent [5,6]. H-radicals have been used in some chemical vapor deposition processes, including those leading to the deposition of amorphous or polycrystalline Si [7,8] and diamond thin films [9,10]. Because an H-radical is more reactive than an H_2_ molecule, the use of H-radicals can lower the Δ*G* of the reduction of SiHCl_3_ and SiCl_4_ [5] and suppress the pyrolysis of SiHCl_3_. For H-radicals to be used in the Siemens method, they should be remotely supplied into the bell jar (reaction chamber) to prevent contaminants from the source gas or synthesized Si from affecting the H-radical generation process. Furthermore, the H-radicals should be generated at a pressure greater than 1 atm (~101 kPa) and transported into the 1 atm reaction chamber, because the conventional Siemens method is operated under ~1 atm to obtain enough Si production rate. When tungsten filament is placed in Siemens chamber, H-radical is not generated due to the formation of silicide. Hydrogen radical should be transported from the other chamber in the pressure higher than ~1 atm. Umemoto et al. [11] reported the transportation of H-radical generated by a filament method at a low pressure of 55 Pa for a generation point and 5.6 Pa for a detection point. The distance between the generating and detection points was 50 cm, which implies an availability of H-radical for the Siemens method. In a previous study, we successfully generated and transported H-radicals using a filament method and reduced SiCl_4_ with the remotely supplied H-radicals under relatively low pressures of approximately 1–10 kPa [6]. The density of hydrogen radical should decrease with an increase of pressure due to three-body recombination (H + H + M (H_2_) → H_2_ + M (H_2_)) [12,13].

In the present study, we generated H-radicals at pressures greater than 1 atm with the filament method and transported them into a 1 atm chamber. In addition, the effect of H-radicals on the reduction of SiCl_4_ at 1 atm was investigated to enable the future application of H-radicals in the Siemens method.

## 2. Experimental details

### 2.1 Apparatus for H-radical generation and transportation

**Figure 1.**
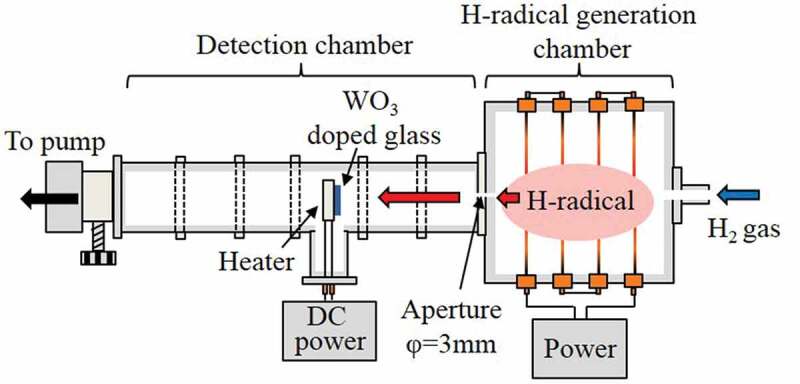
Schematic of an apparatus for H-radical generation and transportation.

A schematic of the apparatus used for the generation and transportation of H-radicals is shown in Figure 1. The apparatus is composed of two chambers: an H-radical generation chamber and a detection chamber. The internal dimensions of the H-radical generation chamber were approximately 30 × 30 × 20 cm^3^. To avoid deactivation of the H-radical by contact with the metal walls of the generation chamber [14], the interior of the chamber was covered by Al_2_O_3_ plates. Four W filaments (diameter: 0.5 mm) were connected in series to electrodes attached to the chamber walls. The length of each W filament was 30 cm; hence, the total length was ~120 cm.

The detection chamber consisted of connected I-shaped NW50 stainless steel tubes and a T-shaped NW50 stainless steel tube. The detection chamber was attached to the H-radical generation chamber; a ⌀3 mm-diameter aperture was created at the joint to enable a pressure difference to be induced between the generation and detection chambers. A ceramic heater (2.5 × 2.5 cm^2^, MS-5, Sakaguchi E.H. VOC Corp., Japan) was installed at the center of the T-shaped tube, and a tungsten oxide (WO_3_)-doped phosphate glass plate (Sankei, HAS-A20, Japan) was placed on the ceramic heater to detect the H-radicals and estimate their density. The distance from the H-radical generation chamber to the detection point could be varied by changing the position of the T-shaped tube. The method used to estimate the H-radical density is described in the next section.

### 2.2. H-radical generation, transportation, and detection

H_2_ gas with a flow rate of 4.2 SLM was introduced into the apparatus from the H-radical generation chamber. The W filaments were then electrically heated using an AC power supply (S-130-40HMD, Yamabishi Denki, Japan) to generate the H-radicals. Upon contact with the heated W filaments, the H_2_ gas was decomposed into H-radicals via the catalytic effect. The generated H-radicals were transported into the detection chamber through the aperture. The pressure in the apparatus was controlled by adjusting the pumping conductance. The W filament temperature was estimated by calculations using previously reported equations [15,16]. Details of the calculations are described in supporting information section S1. Because the H_2_ gas decreases the filament temperature, a larger current than that under a vacuum condition was applied to the W filaments.

The WO_3_-doped phosphate glass was used to detect the H-radicals and to estimate their density. WO_3_-doped glass undergoes a change in color from transparent to dark blue upon reaction with H-radicals; the absorption spectrum of the dark-blue glass shows a peak at ~600 nm [17,18]. To estimate the H-radical density, the transmittance of the WO_3_-doped glass at 600 nm was measured before (*T*_0_) and after (*T*) the sample was exposed to H-radicals. The H-radical density was subsequently estimated on the basis of the relationship between −ln(*T*/*T*_0_) and H-radical density reported by Morimoto et al. [18]. The WO_3_-doped glass was exposed to H-radicals for 1 h with heating at 227°C, 277°C, or 327°C. Details of the method used to estimate the H-radical density are described in supporting information section S2.

The transmittance of WO_3_-doped glass was measured under various gas pressures (10–105 kPa) and at various positions (30–70 cm from the H-radical generation chamber). The temperature of the W filament was also varied from room temperature to ~2000°C by changing the applied current (0–30 A).

### 2.3. Apparatus for H-radical reduction of SiCl_4_

**Figure 2.**
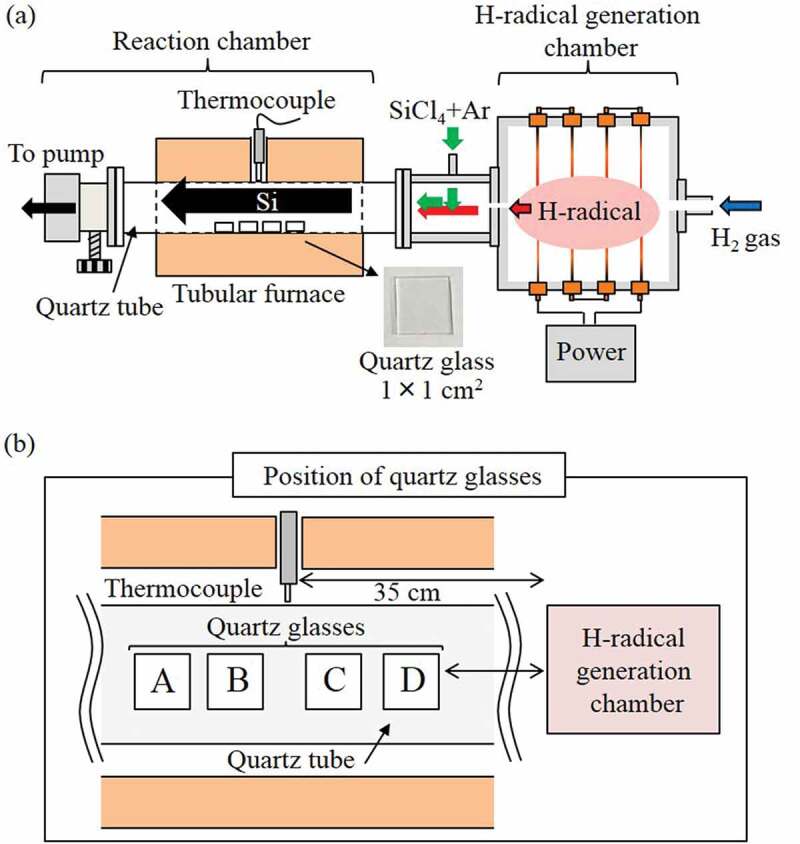
Schematic of (a) the apparatus used for reduction of SiCl_4_ and (b) the position of the quartz glasses.

A schematic of the apparatus used for the H-radical reduction of SiCl_4_ is shown in Figure 2(a). A reaction chamber and a component with a port for the introduction of SiCl_4_ were attached to the H-radical generation chamber in place of the detection chamber used in the previous configuration. A quartz tube (⌀42 mm, length: 50 cm) was used as the reaction chamber. Four quartz glass substrates (dimensions: 1 × 1 cm^2^, MT-WKS100PP01, MONOTECH Co., Ltd, Japan) were placed in the quartz tube as substrates for Si deposition. The position of the quartz glasses is shown in Figure 2(b). The reaction chamber was heated in a tubular furnace. A thermocouple connected to the temperature controller of the tubular furnace was inserted through a hole at the center of the furnace and positioned such that it contacted the outside of the quartz tube.

### 2.4. H-radical reduction of SiCl_4_

The generated H-radicals were transported into the reaction chamber containing SiCl_4_. The SiCl_4_ (6 N, Tri Chemical Laboratories, Japan) was introduced using Ar as a carrier gas. The SiCl_4_ source was maintained at 10°C or 20°C, and the Ar gas flow rate was 15 or 30 sccm. The H-radical and SiCl_4_ mixed gas was heated at ~850°C or 900°C in the reaction chamber for 30 min to reduce the SiCl_4_. Si was deposited inside the quartz tube and onto the quartz glasses.

### 2.5 Characterization

The transmittance of the WO_3_-doped glass substrates was measured by UV–Vis spectroscopy (UV-3150, Shimadzu, Japan). The composition of the obtained substance was analyzed by X-ray diffraction (XRD, Multiflex, Cu-K_α_, 40 kV and 40 mA, Rigaku, Japan) and Raman spectroscopy (PHOTON Design Corp., Japan). The cross-section of the deposited Si was observed by scanning electron microscopy (SEM, JSM-5600LV, JEOL, Japan).

## 3. Results and discussion

### 3.1. Effect of pressure, filament temperature, and transportation distance on H-radical density

**Figure 3.**
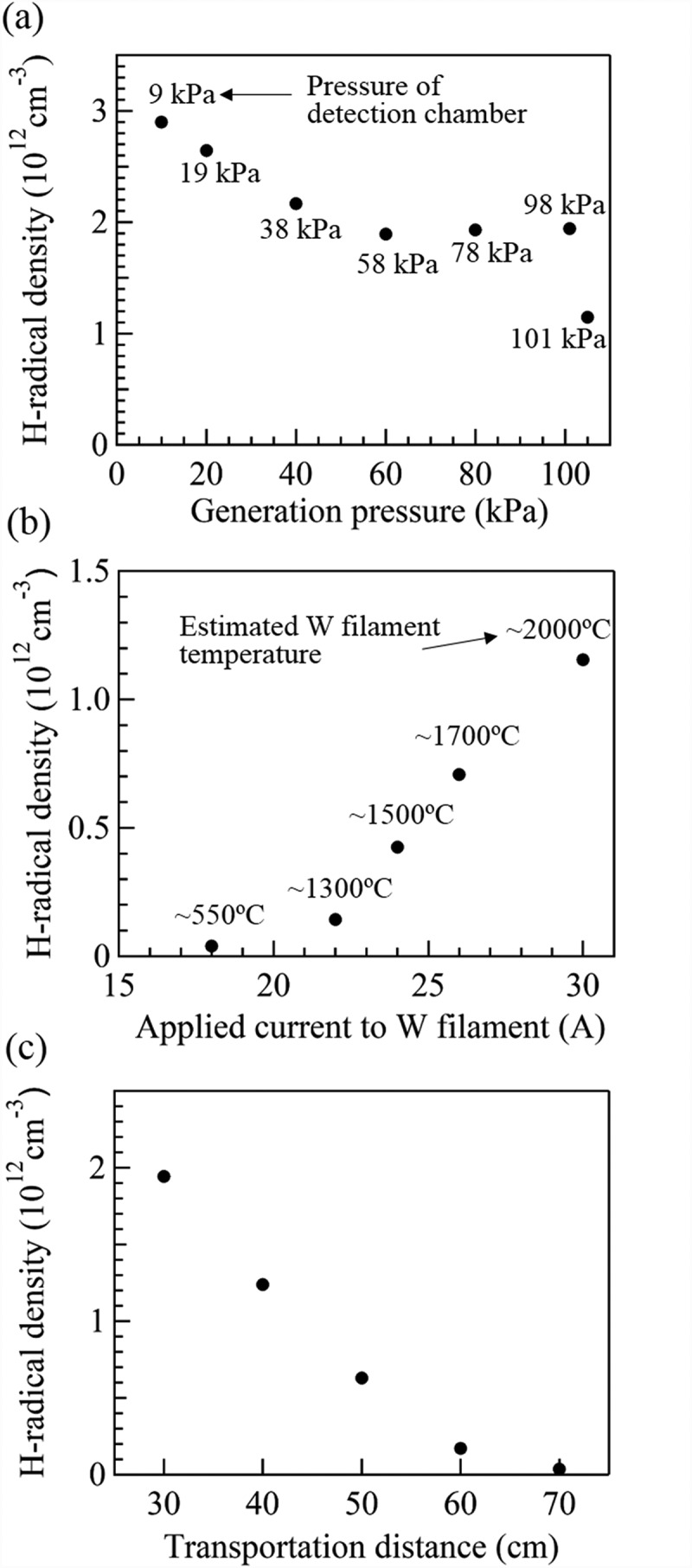
Dependence of the estimated H-radical density on (a) the pressures in the H-radical generation chamber and the detection chamber (the pressures represented in the graph are the pressures in the detection chamber), (b) currents applied to the W filaments (the temperatures indicated in the graph are estimated W filament temperatures), and (c) the transportation distance.

Figure 3(a) shows the estimated density of H-radicals generated under a pressure of 10, 20, 40, 60, 80, 101, or 105 kPa. The pressures in Figure 3(a) represent the pressures in the detection chamber. The difference of pressure between the detection and the generation chamber is listed in Table 1. The H_2_ gas flow rate was 4.2 SLM, the distance from the H-radical generation chamber to the WO_3_-doped glass was 30 cm, and the W filaments were heated to ~2000°C by applying a current of 30 A. The density of the H-radicals generated at 10 kPa (detection chamber: 9 kPa) was estimated to be ~2.9 × 10^12^ cm^−3^. Although the H-radical density decreased with increasing pressure in the generation chamber, a density of ~1.1 × 10^12^ cm^−3^ was attained at a pressure of 105 kPa (>1 atm) in the generation chamber and 101 kPa (= 1 atm) in the detection chamber. The population ratios of H_2_ and H-radical at the detection point were calculated from the H-radical density and H_2_ molecule density. The calculation method is detailed in supporting information section S3. The [H-radical]/[H_2_] population ratio was ~6.5 × 10^−7^ and ~2.3 × 10^−8^ at generation pressures of 10 kPa and 105 kPa, respectively.

**Table 1.** Pressures of generation and detection chambers.

Generation chamber (kPa)	Detection chamber (kPa)	Difference between two chambers (kPa)
10	9	1
20	19	1
40	38	2
60	58	2
80	78	2
101	98	3
105	101	4

The tendency of the H-radical density to decrease with increasing pressure in the generation chamber is in good agreement with previously reported results [12,13]. This trend is attributable to increasing three-body recombination (H + H + M (H_2_) → H_2_ + M (H_2_)) as a result of an increase in the M (H_2_) density at higher pressures [12,13]. In addition, on the basis of Le Châtelier’s principle, the equilibrium of 2 H ⇌ H_2_ shifts rightward to counteract the increase in pressure. This behavior is also attributable to the decrease in the H-radical density with increasing generation pressure [19]. In the case of H-radicals generated using the filament method, Schwarz et al. [10] and Meier et al. [20] have reported H-radical density values of approximately 10^14^–10^15^ cm^−3^. They generated H-radicals at a pressure of approximately 1–10 kPa and detected them near the surface of the filament. Compared with these previously reported values, the H-radical density in the present study was smaller. However, given the experimental conditions used in the present study (i.e., a higher pressure (>1 atm) and longer transportation distance), the obtained H-radical density is reasonable.

Figure 3(b) shows the estimated density of H-radicals generated under various currents applied to the W filaments (18, 22, 24, 26, and 30 A). The temperatures estimated from the filament diameter and the applied current are shown. The H_2_ gas flow rate was 4.2 SLM, and the pressures in the H-radical generation chamber and detection chamber were 105 kPa and 101 kPa, respectively. The distance from the H-radical generation chamber to the WO_3_-doped glass was 30 cm. When the applied current was 18 A (W filament temperature: ~550°C), the transmittance of the WO_3_-doped glass slightly decreased from 82.1% to 78.8%, as shown in **Fig. S2**, corresponding to a low H-radical density of 3.9 × 10^10^ cm^−3^. When the applied current was 22 A (W filament temperature: ~1300°C), the estimated H-radical density increased to ~1.4 × 10^11^ cm^−3^. When the applied current was further increased to 24, 26, and 30 A (filament temperature: ~1500°C, 1700°C, and 2000°C, respectively), the H-radical density rapidly increased to ~4.3 × 10^11^ cm^−3^, ~7.1 × 10^11^ cm^−3^, and ~1.1 × 10^12^ cm^−3^, respectively. These results indicate that H-radicals were effectively generated by the catalytic effect of the W filament at ~1500°C and that the generation rate was enhanced with increasing W filament temperature.

Figure 3(c) shows the estimated density of H-radicals detected at 30, 40, 50, 60, and 70 cm from the H-radical generation chamber. The H_2_ gas flow rate was 4.2 SLM, and the pressures in the H-radical generation chamber and detection chamber were 101 kPa and 98 kPa, respectively. The W filament temperature was ~2000°C (applied current: 30 A). Although the H-radical density exponentially decreased with increasing transportation distance, the H-radicals were successfully detected at distances as far as 50–60 cm from the generation chamber. The flow velocity of H-radical or H_2_ gas in the apparatus is not well understood; hence, the lifetime of the H-radicals could not be estimated. However, because the H-radicals were detected as far as ~50–60 cm from the H-radical generation chamber, the H-radical lifetime may be sufficiently long for the H-radicals to be used in applications where they function as a remote supply.

The decrease of the H-radical density with increasing transportation distance is due to the three-body recombination (H + H + M (H_2_) → H_2_ + M (H_2_)) during the transportation process [12,13]. The decrease of the H-radical density is also attributable to the deactivation of H-radicals by contact with the stainless steel wall of the detection chamber because the stainless steel has a relatively high H-radical recombination probability [21]. Therefore, the coating of the stainless steel wall with Al_2_O_3_ or SiO_2_, both of which have a smaller H-radical recombination probability (Al_2_O_3_: 0.0018 ± 0.0003, SiO_2_: 0.00004 ± 0.00003) [10], minimizes deactivation of the H-radical.

### 3.2. Reduction of SiCl_4_ by H-radicals at relatively low pressures

The H-radical reduction was first conducted at a relatively low pressure because lower pressures tend to lead to greater H-radical densities, as shown in the previous section. The H_2_ gas flow rate was 4.2 SLM, and the pressures in the H-radical generation chamber and reaction chamber were ~3 kPa and ~1.8 kPa, respectively. Before the reduction of SiCl_4_, the H-radical density under the conditions was estimated using the WO_3_-doped glass. The W filaments were electrically heated to ~2000°C by applying 30 A. The WO_3_-doped glass was placed at position B in the quartz tube (~40 cm) from the H-radical generation chamber (Figure 2(b)) and subsequently exposed to H-radicals for 1 h with heating at 327°C (600 K). Figure S3 shows the transmittance spectra of the WO_3_-doped glass before and after it was exposed to H-radicals. The transmittance at 600 nm decreased from ~83.5% (original) to 14.1% after the exposure to H-radicals, which gave an estimated H-radical density of ~1.3 × 10^12^ cm^−3^. The [H-radical]/[H_2_] population rate was calculated to be ~2.8 × 10^−6^.

**Figure 4.**
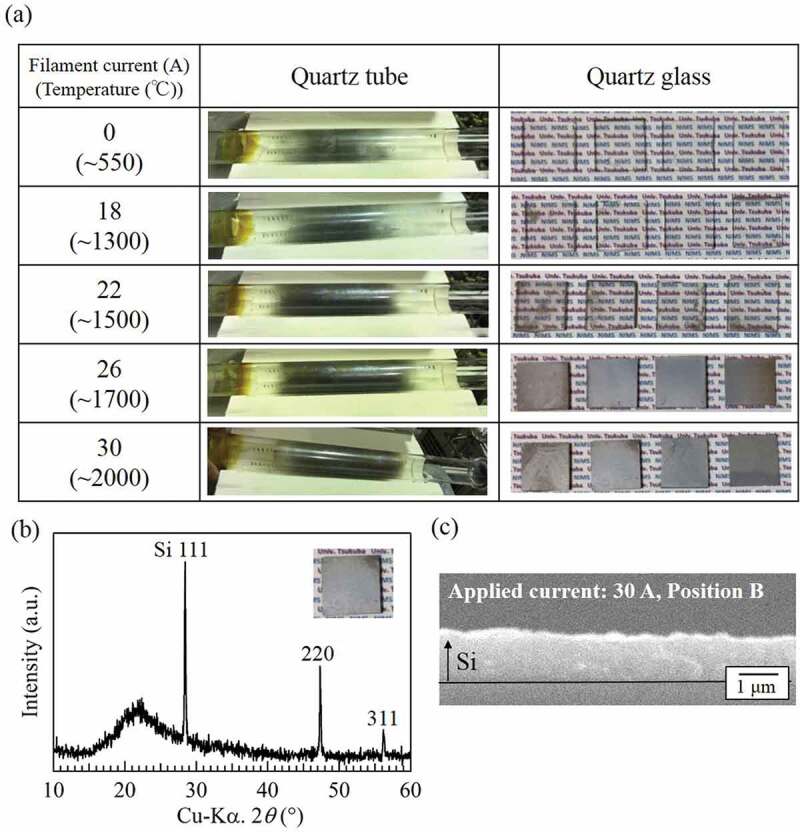
(a) Appearance of quartz tubes and quartz glasses after the reduction of SiCl_4_. (b) XRD pattern and (c) cross-sectional SEM image of the quartz glass placed at the position B during the experiment with an applied current of 30 A. The pressure in the H-radical generation chamber and the reaction chamber were ~3 kPa and ~1.8 kPa, respectively.

The H-radical reduction of SiCl_4_ was subsequently carried out. The temperature of the W filament was varied by changing the applied current. The temperature of the reaction chamber was 900°C, the amount of SiCl_4_ introduced was ~330 μmol/min, and the reaction time was 30 min. Figure 4(a) shows photographs of the quartz tubes and quartz glasses after the experiments. When only H_2_ gas was used (current applied to W filaments: 0 A, filament temperature: room temperature (RT)), the dark-gray substance was confirmed to have only coated the inside of the quartz tube. Similar results were obtained with an applied current of 18 A. When the applied current was increased to 22 A (filament temperature: ~1300°C), the amount of dark-gray substance in the quartz tube increased and the dark-gray substance was confirmed to have coated the quartz glasses. When the applied current was further increased to 26 A and 30 A (filament temperature: ~1700°C and ~2000°C), the amount of dark-gray substance on both quartz tubes and glasses increased substantially. The composition of the gray substance on the quartz glasses was analyzed by XRD. Figure 4(b) shows the XRD pattern for the quartz glass placed at position B in the experiment with an applied current of 30 A. Three peaks corresponding to Si are observed at 2*θ ≈* 28.4°, 47.3°, and 56.2°, indicating that the gray substance is Si.

As shown in Figure 3(b), the H-radical density increased rapidly under a pressure of 1 atm when the applied current was ~22 A or greater (filament temperature: ~1300°C). From the results, we speculated that the H-radical density at the generation pressure of 3 kPa also increased at an applied current of ~22 A or greater, similar to the case of 1 atm. Therefore, Si was deposited onto the glasses in cases where the filament current was greater than 22 A, as shown in Figure 4(a). We confirmed the reduction of SiCl_4_ by detecting the partial pressure of SiCl_4_ via quadruple mass measurement when the H-radicals were supplied remotely. These results suggest that the generated H-radicals promoted the reduction of SiCl_4_ to Si.

Figure 4(c) shows a cross-sectional SEM image of the quartz glass placed at position B during the experiment with an applied current of 30 A. The thickness of the deposited Si was ~1 μm. To estimate the amount of SiCl_4_ reduced to Si, we calculated the mole number of Si deposited onto the quartz tube. The volume of deposited Si was calculated to be ~2.0 × 10^−2^ cm^3^ by multiplying the inside diameter of the quartz tube (3.9 cm), the circumference (3.14), the length of the area coated with Si on the quartz tube (16 cm), and the thickness of the deposited Si (1 μm). The obtained volume value was then multiplied by the mass of Si and divided by the atomic weight of Si. As a result, the mole number of deposited Si was estimated to be ~1.6 × 10^−3^ mol. Because the amount of SiCl_4_ supplied during the experiment was ~1.0 × 10^−2^ mol (330 μmol/min × 30 min), ~10% of the supplied SiCl_4_ was reduced to Si when a current of 30 A was applied to the W filaments.

In addition, the deposition rate of Si with an applied current of 30 A was also calculated to be ~2 μm/h from the thickness of the deposited Si. Actually, Theuerer et al. [22], who also investigated the H_2_ reduction of SiCl_4_, reported a greater deposition rate of Si (~300 μm/h) than that observed in the present study. The smaller deposition rate in the present study is ascribed to a difference in the SiCl_4_ supply. In the Theuerer’s report, the mole fraction of supplied SiCl_4_ was 0.1 in 1.0 L/min H_2_ gas to achieve a deposition rate of ~300 μm/h. They also reported that the deposition rate tended to decrease with decreasing SiCl_4_ supply. In the present study, the mole fraction of SiCl_4_ in H_2_ gas was calculated to be ~1.8 × 10^−3^on the basis of the flow rates of SiCl_4_ (~330 μmol/min) and H_2_ gas (~0.188 mol/min, 4 SLM). This amount is substantially smaller than that used by Theuerer et al., and our smaller SiCl_4_ supply may have resulted in the relatively lower deposition rate of Si. Thus, the deposition rate can likely be increased through the optimization of the SiCl_4_ supply.

### 3.3. Reduction of SiCl_4_ by H-radicals at 1 atm

**Figure 5.**
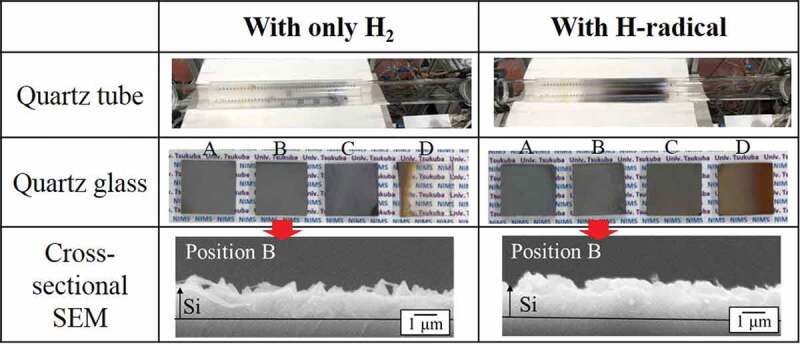
Appearances of quartz tubes and quartz glasses after the reduction of SiCl_4_ with H_2_ and H-radicals at 900°C, and cross-sectional SEM images of the quartz glasses placed at position B. The pressures in the H-radical generation chamber and the reaction chamber were ~105 kPa and ~101 kPa, respectively.

The reduction of SiCl_4_ by H-radicals was carried out at a reaction pressure of 1 atm (~101 kPa). For the H-radical reduction of SiCl_4_ at a reaction pressure of ~101 kPa, the H_2_ gas flow rate and the pressures in the H-radical generation chamber and the reaction chamber were the same as those described in section 3.2. The reaction chamber was heated to 900°C, and the amount of SiCl_4_ introduced was decreased to ~104 μmol/min, because H-radical density was lower in atmospheric pressure. Figure 5 shows the appearance of the quartz tubes and quartz glasses after the reduction of SiCl_4_ by H_2_ or H-radicals. Cross-sectional SEM images of the quartz glasses placed at position B during the experiment are also shown.

Under the 1 atm condition, Si film was deposited onto the quartz glasses at positions A, B, and C, even when only H_2_ gas was used (applied current: 0 A, filament temperature: RT); this result is attributed to the larger numbers of density of SiCl_4_ and H_2_ in the reaction chamber due to lower flow velocity than those under a pressure of ~1.8 kPa. Actually, the thickness of Si film was thicker than 1 μm in Figure 4. However, the quartz tube remained transparent after the experiment. By contrast, when H-radicals were generated, the quartz tube became opaque because of the deposited Si, which indicates an increase in the amount of Si produced by the H-radicals. The thickness of the Si deposited with only H_2_ gas was ~1.3 μm, while the thickness slightly increased to ~1.5 μm when deposition was carried out with H-radicals. Although this difference in thickness is smaller than that under a pressure of ~1.8 kPa because of the lower H-radical density, the increase in the amount of Si produced by the H-radicals at 1 atm was confirmed. Taking a lower density of H-radical in ~1 atm into account, we considered that H-radical with longer lifetime must react with SiCl_4_ several times.

For the quartz glass placed at position D, Si was hardly observed when only H_2_ gas was used. Because position D is farther from the center of the tubular furnace than positions B and C, the temperature at position D is lower. As a result, SiCl_4_ was not effectively reduced at position D, and Si was not formed. A similar situation occurred at position A; however, because the H_2_ and SiCl_4_ mixed gas passes through positions B and C (higher-temperature area) before reaching position A, Si can be produced and deposited onto the quartz glass. By contrast, with the H-radicals, the quartz glass at position D was completely covered with Si, implying that the SiCl_4_ reduction by H-radicals proceeded at lower temperatures than that by H_2_ gas.

**Figure 6.**
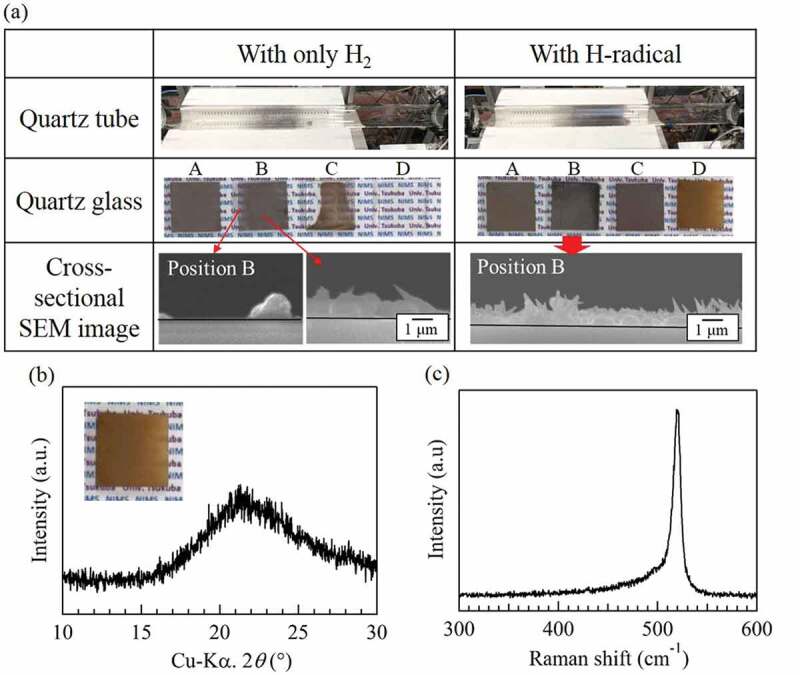
(a) Appearances of the quartz tubes and quartz glasses after the reduction of SiCl_4_ with H_2_ and H-radicals at 850°C, and cross-sectional SEM images of the quartz glasses placed at positions A–D. (b) XRD pattern and (c) Raman spectrum of the quartz glass placed at position D during the H-radical reduction of SiCl_4_. The pressures in the H-radical generation chamber and the reaction chamber were ~105 kPa and ~101 kPa, respectively.

Figure 6(a) shows the appearance of the quartz tubes and quartz glasses after the reduction of SiCl_4_ with H_2_ and H-radicals at 850°C. The cross-sectional SEM images of the quartz glasses at position B are also included. For the condition with only H_2_, the amount of Si deposited onto the quartz glasses with H_2_ gas at 850°C decreased dramatically compared with that deposited at 900°C. Although Si was deposited onto the quartz glasses at positions A and B, some parts of the quartz glasses were not covered, as shown in the cross-sectional SEM images. In addition, almost no Si was deposited on the quartz glasses at positions C and D. By contrast, the quartz glasses at positions A, B, and C were completely covered with Si when the reduction was carried out with H-radicals. This indicates an increase in the amount of Si produced by H-radicals. These results also suggest that H-radicals effectively promote the reduction of SiCl_4_ even at 1 atm. The thickness of the deposited Si on the quartz glass at position B was ~1 μm. Some needle-shaped Si was confirmed on the surface, although the formation mechanism of this Si is not yet well understood.

The substance formed on the quartz glass at position D exhibited a brown color that differed from that of the substances formed at the other positions. To identify this substance, we analyzed it by XRD and Raman spectroscopy. Figure 6(b,c) shows the XRD pattern and Raman spectrum of the brown substance, respectively. A strong peak for Si was not observed in the XRD pattern; however, the XRD pattern did show a weak reflection at ~28.4°. In the case of the Raman spectrum, a strong peak at ~520 cm^−1^ corresponding to crystalline Si was confirmed, but the bottom of the peak was broadened from ~510 to 450 cm^−1^. Because amorphous Si is known to have a broad peak at ~480 cm^−1^ in its Raman spectrum [23], the brown substance may be amorphous Si.

## 4. Conclusions

In this study, we demonstrated the generation and transportation of H-radicals at pressures greater than 1 atm to enable H-radicals to be used in the Siemens method for the production of Si. Moreover, the effect of H-radicals on the reduction of SiCl_4_ was also investigated. In the case of the generation and transportation of H-radicals, an estimated H-radical density of ~1.1 × 10^12^ cm^−3^ was attained under a generation chamber pressure of 105 kPa (>1 atm) and a detection chamber pressure of 101 kPa (1 atm), although the H-radical density decreased with increasing pressure. In addition, H-radicals were detected even at positions ~50–60 cm from the generation chamber, which indicates that the H-radicals exhibit a sufficiently long lifetime to be used in remote-supply applications. For the reduction of SiCl_4_, the amount of Si produced from the reduction of SiCl_4_ was substantially increased through the use of H-radicals at a relatively low reaction pressure of ~1.8 kPa. Even at a reaction pressure of 1 atm (~101 kPa), the amount of Si produced was increased by the use of H-radicals. The H-radicals were applied to the Siemens method and were found to improve the Si yield.

## Supplementary Material

Supplemental Material
