# Supplementary material for: Effective silicon production from SiCl4 source using hydrogen radicals generated and transported at atmospheric pressure
Source: Sci Technol Adv Mater. 2020 Jul 27;21(1):482–91. doi: 10.1080/14686996.2020.1789438 (PMC7476485; doi:10.1080/14686996.2020.1789438)
Supplement: Supplemental Material [file TSTA_A_1789438_SM1606.pdf]

Supporting information

Effective Silicon production from $SiCl_4$ source by using hydrogen radical generated and transported at atmospheric pressure

Yuji Okamoto[1,2], Masatomo Sumiya[1*], Yuya Nakamura[1,2], and Yoshikazu Suzuki[3]

[1] Widegap Materials group, National Institute for Materials Science, 1-1 Namiki, Tsukuba, Ibaraki 305-0044, Japan.

[2] Graduate School of Pure and Applied Sciences, University of Tsukuba, Ibaraki 305-8573, 1-1-1 Tennodai, Tsukuba, Ibaraki 305-8573, Japan.

[3] Faculty of Pure and Applied Sciences, University of Tsukuba, 1-1-1 Tennodai, Tsukuba, Ibaraki 305-8573, Japan.

*E-mail: SUMIYA.Masatomo@nims.go.jp

Information Classification: General

S 1. Calculation of filament temperature

The W filament temperature was estimate by the calculation using the following reported equation (**eq. S1**[i)]),

$$I^2 \times \rho \,/\, (\pi d^2 / 4) = \varepsilon\sigma T^4 \times \pi d \qquad \textbf{(eq. S1)}$$

where I is an applied current, ρ is a resistivity, d is a diameter of the filament, ε is an emissivity, σ is Stefan Boltzmann constant, and T is the filament temperature. The resistivity of tungsten was calculated by the following reported equation (**eq. S2**[ii)]).

$$\rho = -1.72573 + 2.14350 \times 10^{-2}T + 5.74811 \times 10^{-6}T^2 - 1.13698 \times 10^{-9}T^3 + 1.1167 \times 10^{-13}T^4 \qquad \textbf{(eq. S2)}$$

The ε of tungsten was calculated using the following equation (**eq. 3.5**[i)]).

$$\varepsilon = -3.7 \times 10^{-8}T^2 + 0.00026T - 0.112 \qquad \textbf{(eq. S3)}$$

Since H_2 gas decreases the filament temperature, larger currents than that under the vacuum condition were applied to the W filaments.

S 2. Estimation method of H-radical density

The H-radical density was estimated using transmittance of the WO_3 doped glass before and after the H-radical exposure. **Figure S1(a)** shows the relationship between -ln(T/T_0) and H-radical density reported by Morimoto et al..[iii)] To make the **Fig. 1(a)**, the WO_3 doped glasses were exposed to H-radical for 1 h with annealing at 327°C (600 K). Since the transmittance change by the H-radical exposure also depends on the annealing temperature, they also showed the relationship between -ln(T/T_0) and annealing temperature during the exposure to H-radical as shown in **Fig. S1 (b)**. From the **Fig. S1(b)**, the ln(T/T_0) at 327°C (600 K) was roughly 1.96 and 1.36 times larger than those at 227°C (500 K) and 277°C (550 K), respectively.

In this study, the transmittances after the H-radical exposure at 600 nm were too low (less than 0.1 %) with some experimental conditions. Therefore, the annealing temperature of WO_3 doped glass was varied in 227°C, 277°C and 327°C. When the annealing temperature of 227°C

Information Classification: General

or 277°C was used, 1.96 or 1.36 was multiplied to the obtained -ln(T/T_0) to convert them into that at 327°C. Then, the H-radical density was estimated using calculated -ln(T/T_0) and **Fig. S1(a)**.

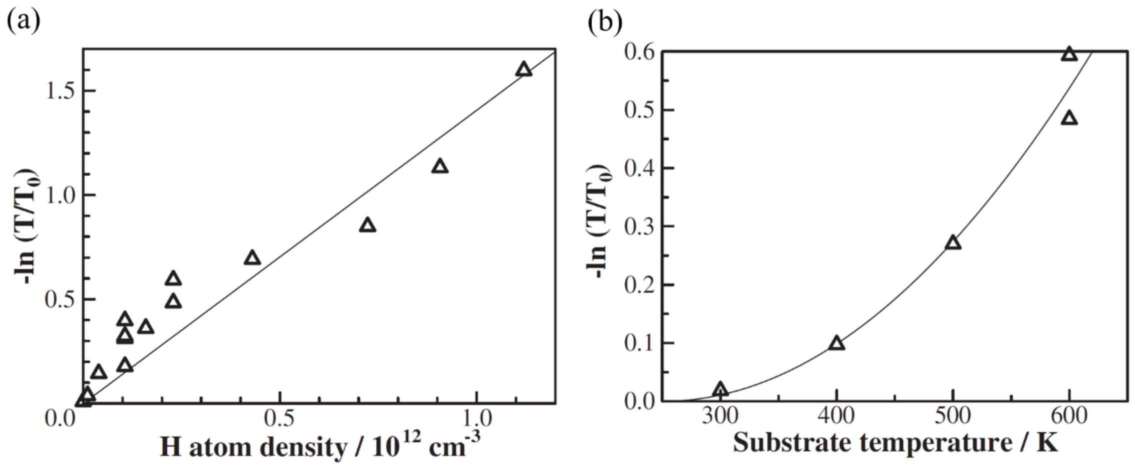

Figs. S1 (a) Relationship between -ln(T/T_0) and H-radical density. The WO_3 doped glass was annealed at 327°C and exposed to H-radical for 1 h. (b) Relationship between -ln(T/T_0) and annealing temperature of WO_3 doped glass. The exposure time to H-radical was 1 h. These figures were reported by Morimoto et al..[iii)]

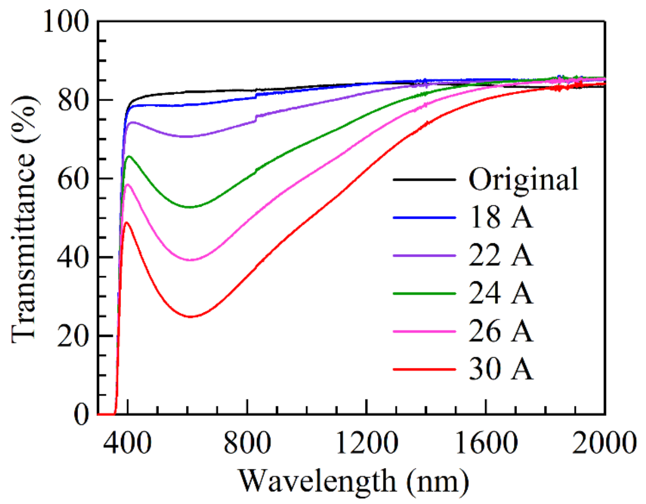

Fig. S2 Transmittance spectra of the WO_3 doped glass before and after the exposure to H-radical generated with various applied current to W filaments.

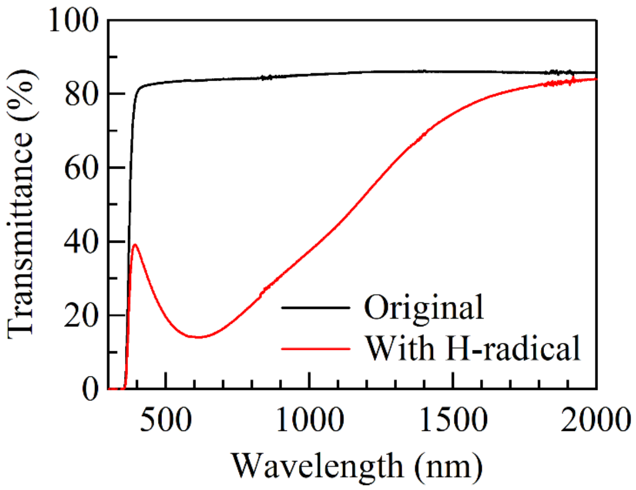

Fig. S3 Transmittance spectra of WO_3 doped glass before and after the exposure to H-radical at the pressures of ~3 kPa in the H-radical generation chamber and ~1.8 kPa in the reaction chamber.

References

i) K. Tanuma, H. Ohba, T. Shibata, *JAERI-Tech*, 99-050 (1999).

ii) P. D. Desal, T. K. Chu, H. M. James, C. Y. Ho, J. Phys. Chem. Ref. Data*,* **13**, 1069 (1984).

iii) T. Morimoto, H. Umemoto, K. Yoneyama, A. Masuda, H. Matumura, K. Ishibashi, H. Tawarayama, H. Kawazoe, Jpn. J. Appl. Phys., **44**, 732 (2005).

Information Classification: General
